# Effect of the OPTIMAL programme on self-management of multimorbidity in primary care: a randomised controlled trial

**DOI:** 10.3399/bjgp20X714185

**Published:** 2020-03-09

**Authors:** Lynn O’Toole, Deidre Connolly, Fiona Boland, Susan M Smith

**Affiliations:** Discipline of Occupational Therapy, School of Medicine, Trinity College Dublin, St James’s Hospital, Dublin.; Discipline of Occupational Therapy, School of Medicine, Trinity College Dublin, St James’s Hospital, Dublin.; Data Science Centre, Royal College of Surgeons in Ireland, Dublin.; HRB Centre for Primary Care Research, Department of General Practice, Royal College of Surgeons in Ireland, Dublin.

**Keywords:** multimorbidity, occupational therapy, randomised controlled trial, self-management

## Abstract

**Background:**

Effective primary care interventions for multimorbidity are needed.

**Aim:**

To evaluate the effectiveness of a group-based, 6-week, occupational therapy-led self-management support programme (OPTIMAL) for patients with multimorbidity.

**Design and setting:**

A pragmatic parallel randomised controlled trial across eight primary care teams in Eastern Ireland with 149 patients with multimorbidity, from November 2015 to December 2018. Intervention was OPTIMAL with a usual care comparison.

**Method:**

Primary outcomes were health-related quality of life (EQ-5D-3L) and frequency of activity participation (Frenchay Activities Index [FAI]). Secondary outcomes included independence in activities of daily living, occupational performance and satisfaction, anxiety and depression, self-efficacy, and healthcare utilisation. Complete case linear regression analyses were conducted. Age (<65/≥65 years) and the number of chronic conditions (<4/≥4) were explored further.

**Results:**

A total of 124 (83.2%) and 121 (81.2%) participants had complete data at immediate and 6-month post-intervention follow-up, respectively. Intervention participants had significant improvement in EQ-VAS (visual analogue scale) at immediate follow-up (adjusted mean difference [aMD] = 7.86; 95% confidence interval [CI] = 0.92 to 14.80) but no difference in index score (aMD = 0.04; 95% CI = −0.06 to 0.13) or FAI (aMD = 1.22; 95% CI = −0.84 to 3.29). At 6-month follow-up there were no differences in primary outcomes and mixed results for secondary outcomes. Pre-planned subgroup analyses suggested participants aged <65 years were more likely to benefit.

**Conclusion:**

OPTIMAL was found to be ineffective in improving health-related quality of life or activity participation at 6-month follow-up. Existing multimorbidity interventions tend to focus on older adults; preplanned subgroup analyses results in the present study suggest that future research should target younger adults (<65 years) with multimorbidity.

## INTRODUCTION

Individuals with multimorbidity, the presence of ≥2 chronic conditions, have poorer health outcomes, higher health service utilisation, and higher healthcare costs.^1^^,^^2^ The 2016 updated Cochrane review of interventions for multimorbidity in primary care^3^ found limited evidence on effectiveness. Included interventions predominately centred on care organisation such as case management or patient-oriented interventions, for example, patient education interventions. The review concluded that previous interventions focused predominantly on people with defined comorbid conditions or on multimorbidity in patients aged >65 years, and recommended a focus on risk factors common across comorbid conditions or generic outcomes such as daily functioning.^3^ In 2018, the largest randomised controlled trial (RCT) of a multimorbidity intervention, the 3D study,^4^ examined the effect of general practice-based 6-monthly patient multidisciplinary reviews of the dimensions of drugs, depression, and health, based on multimorbidity guidelines. It found no effect on health-related quality of life (HRQoL) but did report significant improvements in patients’ experience of care.

Self-management interventions aim to maximise physical and psychosocial functioning by providing individuals with skills to manage symptoms, treatments, and the psychosocial consequences of living with a chronic condition.^5^^,^^6^ The clinical and cost-effectiveness of such interventions for multimorbidity remain unclear.^7^ Studies of the popular peer-led Stanford chronic disease self-management programme have produced modest effects when delivered in settings outside of the US.^8^ The Medical Research Council framework for complex interventions^9^ was used to develop and pilot OPTIMAL, a professionally-led 6-week group self-management support programme for multimorbidity.^10^^,^^11^ The OPTIMAL programme is underpinned by self-efficacy theory, focusing on topics of concern to those with multimorbidity, and is professionally led by primary care occupational therapists, because of the profession’s focus on function, with input from a physiotherapist and pharmacist. Further details of the OPTIMAL programme content and delivery are outlined in Boxes 1 and 2. A pilot RCT of the OPTIMAL programme provided preliminary evidence that the programme significantly improved frequency of activity participation in instrumental activities of daily living, self-efficacy, and HRQoL immediately post-intervention.^11^

**Box 1.** OPTIMAL programme elements

**Theory:** Self-efficacy theory incorporating influencers including performance accomplishments, vicarious learning, social/verbal persuasion, reinterpretation of physiological and emotional states
**Format:** Group-based programme delivered over 6 consecutive weeks; 2.5 hour session with tea/coffee break
**Location:** Primary care centres or community resource centres
**Mode of delivery:** Educational (includes participant interaction and discussion) and goal-setting components
**Facilitators:** Health Service Executive (HSE) primary care occupational therapists with input from physiotherapist and pharmacist
**Educational component:** Week 1: Introduction to self-management, activity and health, and goal-setting Week 2: Fatigue management and healthy eating Week 3: Maintaining physical activity Week 4: Maintaining mental wellbeing Week 5: Managing medications Week 6: Communication and programme review
**Goal-setting component:** Overall programme goals set in Week 1. Weekly goal-setting and review
**Resources:** Participant booklet, relaxation CD, information on local resources, HSE health promotional resources, such as exercise booklets, get active your way, healthy eating, and information on generic medicines and mental health

**Box 2.** OPTIMAL programme weekly content

**Week 1** Introduction to group, self-management, and programme overview Impact of multimorbidity on activity Explanation of goal setting Set overall programme goals
**Week 2** Fatigue management principles Using fatigue strategies in daily activities Healthy eating principles Healthy eating challenges and small changes Set individual weekly goals
**Week 3** Benefits of exercise Exploring physical activity levels Keeping fit at home and in the community Weekly goal review Set individual weekly goals
**Week 4** Triggers and signs of stress Strategies to maintain mental health Relaxation strategies Sleep hygiene Weekly goal review Set individual weekly goals
**Week 5** Understanding medications Barriers to managing medication Medication management strategies and products Weekly goal review Set individual weekly goals
**Week 6** Communicating with health professionals Communicating with families Reflecting on past communication difficulties and new solutions Programme review Weekly goal review Community resources Presentation of certificate

The aim of the present study was to conduct a definitive RCT to evaluate the effectiveness of the OPTIMAL programme in improving HRQoL and frequency of activity participation, and to test its sustainability after programme completion, as per Stage III of the Medical Research Council framework.^9^

**Table:** How this fits in

Existing interventions for multimorbidity are associated with little benefit to quality of life. The 2016 updated Cochrane review of interventions for multimorbidity concluded that interventions may be more effective if they focus on risk factors common across comorbid conditions or generic outcomes, such as daily functioning. The OPTIMAL programme, a 6-week, professionally-led, self-management support group intervention, aimed to have a specific focus on function and issues relevant to multimorbidity. The present study showed that, overall, there was no evidence the intervention had an effect on quality of life or functioning at 6-month follow-up. There remains a need to develop effective interventions to improve outcomes for patients with multimorbidity in primary care.

## METHOD

### Design

A pragmatic parallel two-arm RCT was reported following CONSORT guidelines for the design, conduct, and analysis of RCTs.^12^

### Setting

The study was carried out in the Irish primary care health system in the greater Dublin region. Ireland has a mixed public and private primary healthcare system, with one-third of the population entitled to free primary care based on low income through the General Medical Services Scheme. Primary care teams typically include GPs and practice nurses who are independent contractors, and allied health practitioners including community nurses, occupational therapists, physiotherapists, dietitians, and social workers, all of whom are employees of the public health system, that is, the Health Service Executive. Primary care in Ireland remains underdeveloped and fragmented;^13^^–^^15^ a process evaluation was also conducted alongside the OPTIMAL trial to evaluate its implementation within existing primary care services in Ireland and will be reported separately.

### Participants and recruitment

Participants with multimorbidity were recruited through primary care team members and self-referral across eight Health Service Executive primary care areas in which participating occupational therapists were based. Recruitment and intervention delivery was conducted over four sequential time blocks, with two primary care team areas in each time block. Referring clinicians were informed about the study via post, email, and presentations at primary care team meetings. Referrals were forwarded to a gatekeeper in each area’s occupational therapy department who contacted referred patients 7–10 days after referral to confirm their participation.

The following participant inclusion criteria were applied: age ≥40 years; ≥2 chronic conditions; ≥4 repeat medications; and an ability to travel to the centre where the intervention would be delivered. These criteria were the same as those used in the exploratory RCT, which proved to be effective, with the exception of age.^11^ The age limit of ≥40 years was chosen because multimorbidity is relatively uncommon in patients younger than this and to facilitate targeted recruitment.^16^^,^^17^ A broad, inclusive, and commonly used definition of ≥2 chronic conditions was used. The World Health Organization’s definition of chronic diseases as health problems that require ongoing management over a period of years or decades was used.^18^ The inclusion criteria of ≥4 repeat medications was included to identify a group in the broader multimorbidity population that is at increased risk of poor health outcomes and more likely to benefit from an intervention.^3^ Written informed consent was obtained from all trial participants. The trial ran from November 2015 to December 2018.

### Intervention and control groups

Following baseline data collection for each site (area), participants were individually randomised into intervention (OPTIMAL programme) or control (waiting list continuing to receive care as usual) groups. Randomisation and allocation was carried out remotely by an independent statistician. Randomisation was performed using Stata (version 14), was stratified by sex, and random permutated blocks of size 2 and 4 were used. Couples who were recruited were randomised as a unit to avoid contamination. The independent statistician informed therapists at each site of participant allocation and therapists in turn informed participants of their allocation by telephone 1–2 weeks before the intervention began.

### Intervention

Full details of the OPTIMAL programme have been published previously and the programme content is summarised in Boxes 1 and 2.^10^^,^^11^ Before programme delivery, occupational therapists received a half-day of training and a facilitator manual to standardise programme delivery and maintain intervention fidelity.

### Outcomes

Outcomes were chosen to reflect the intervention’s theoretical underpinnings and based on the previous OPTIMAL pilot studies.^10^^,^^11^ Outcomes were collected immediately post-intervention (primary outcome measures only) and 6 months post-intervention.

Baseline assessments were conducted via interview with occupational therapists in each site. Immediately post-intervention, intervention and control participants self-completed primary outcomes by postal survey (in a 3-week period of intervention completion). All 6-month follow-up (post-intervention completion) assessments were collected via interviews, with a researcher blinded to participant allocation and a record of broken blinding was maintained. Because of the nature of the intervention, it was not possible to blind participants to their group allocation. Data collection for control participants was matched to intervention participants in the same time block to ensure an equal length of follow-up.

Two primary outcome measures were used: HRQoL (measured using the EQ-5D-3L) and frequency of activity participation (measured using the Frenchay Actvities Index [FAI]).^19^^,^^20^ The EQ-5D-3L comprises two parts, the descriptive system and a visual analogue scale (EQ-VAS). The descriptive system consists of five dimensions including mobility, self-care, usual activities, pain/discomfort, and anxiety/depression, each of which is rated on three severity levels. The EQ-VAS is a vertical visual analogue scale whereby participants rate their perceived health status from 0 (worst imaginable health) to 100 (best imaginable health). The EQ-5D-3L descriptive system can be converted to an index score based on societal preferences for health states.^21^ Secondary outcome measures included the Nottingham Extended Activities of Daily Living scale,^22^ the Stanford Chronic Disease Self-Efficacy 6-item Scale (SEMCD),^23^ the Hospital Anxiety and Depression Scale (HADS),^24^ and the Canadian Occupational Performance Measure (COPM).^25^ Self-reported healthcare utilisation, including GP visits, emergency department visits, outpatient appointments, hospital admissions, and nights spent in hospital was collected for 6 months before baseline data collection and 6 months post-intervention (see Supplementary Table S1 for additional details of the outcomes and scoring interpretation).

### Sample size calculation

A sample size of 200 participants was calculated, using pilot trial data, based on 90% power to detect a clinically relevant change in both primary outcome measures at a 0.05 significance level and to allow for 30% loss to follow-up (see Supplementary Box S1 for details). However, difficulties with recruitment resulted in revision of the sample size using interim trial baseline data (*n* = 108) and 80% power. Furthermore, retention was 10% higher at follow-up than originally anticipated. In the EQ-VAS, improvements of 14 points have been reported as representing a large effect size.^26^ Interim mean EQ-VAS baseline scores were 59.1 (standard deviation [SD] 20.3). To improve a baseline EQ-VAS score of 59.1 by 14 points, with 80% power, required a total sample size of 68 (*n* = 34 per group). Improvements of 4 points in FAI total scores have been reported as clinically significant.^27^ Interim mean FAI baseline scores were 25.3 (SD 7.5). To improve a baseline FAI score by 4 points, with 80% power, a sample size of 114 in total (*n* = 57 per group) was required. The revised sample size calculation indicated that the study required 144 participants, which incorporated a 20% loss to follow-up.

### Data analysis

All results were analysed using Stata (version 14). For primary and secondary outcomes, the primary analysis was intention to treat, including all randomised participants, all retained in the group to which they were allocated, and using complete case analyses. All analyses used multiple linear regression models, with results presented as point estimates (mean differences [MD]), 95% confidence intervals (CI), and *P*-values. Statistical significance at *P*<0.05 was assumed. Adjusted and unadjusted models were explored. As per the European Medicines Agency recommendations of adjusting for stratification variables and variables known *a priori* to be related to outcome, models were adjusted for sex (stratification variable), baseline scores, area, number of conditions, and age. The intervention was group based (by area). Given the small number of areas, however, all models were adjusted for area by including area as a fixed effect. Further analyses were explored by adjusting for differences in marital status at baseline but no differences were seen.^28^

A pre-planned secondary per protocol analysis was conducted, excluding those randomised who did not receive the intervention (non-adherence was defined as attending <3 OPTIMAL sessions), based on previous studies.^29^ Furthermore, pre-planned subgroup analyses based on previous literature^3^ evaluated the effects of age (<65 and ≥65 years) and the number of chronic conditions present (<4 and ≥4) by adding interactions with allocation to the models. Such analyses can be useful in individualising patient care.^30^ A sensitivity analysis was conducted excluding the couples recruited (see Supplementary Tables S2 and S3 for details).

## RESULTS

**Figure 1.**
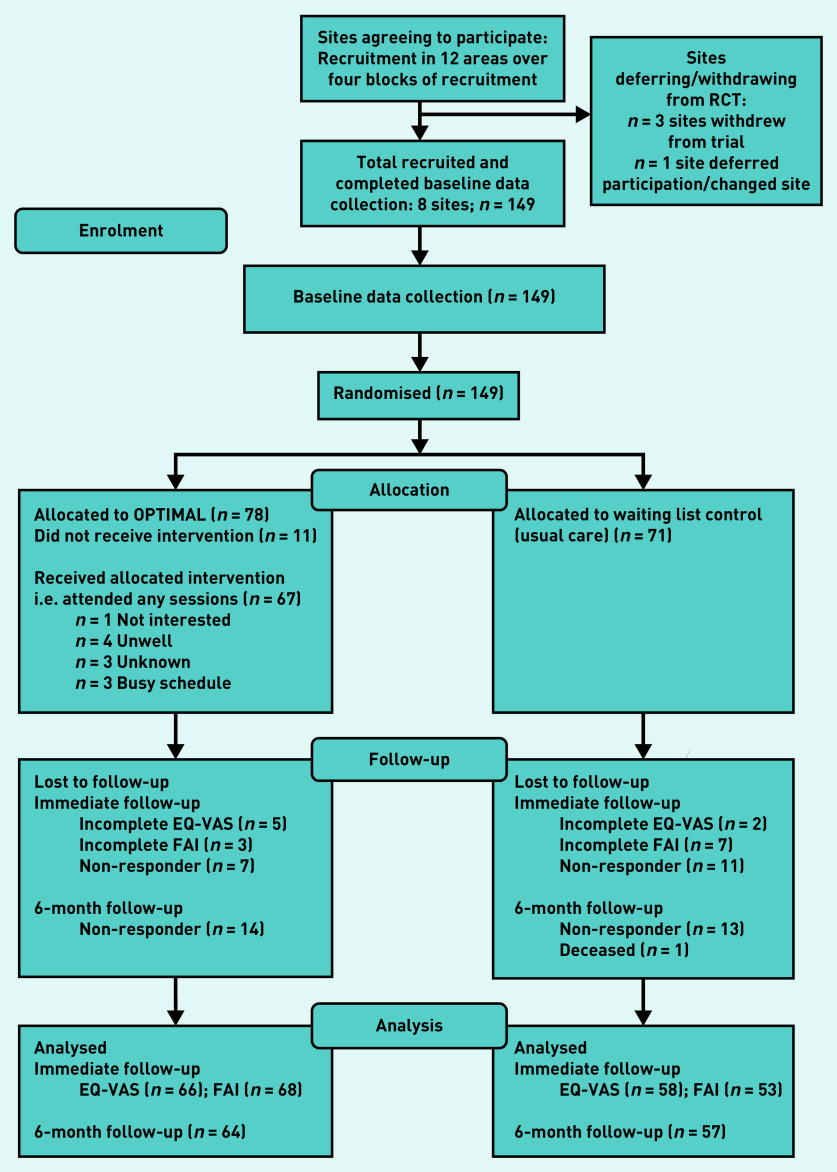
***CONSORT flow diagram.*** ***EQ-VAS = EQ visual analogue scale. FAI = Frenchay Activities Index. RCT = randomised controlled trial.***

Participants were recruited between February 2016 and February 2018. In total, 149 participants consented and completed baseline data collection (Figure 1). A total of 124 (83.2%) and 121 (81.2%) participants had complete data at immediate and 6-month follow-up, respectively. Most of the intervention group attended ≥3 sessions (*n* = 59; 75.6%). Table 1 summarises participants’ baseline characteristics. Table 2 presents adjusted intention-to-treat analyses for primary and secondary outcomes. Unadjusted intention-to-treat analyses (see Supplementary Table S4 for details) and per protocol analyses (see Supplementary Tables S5 and S6 for details) of outcomes were conducted.

**Table 1.** Summary of participants’ characteristics at baseline

**Characteristic**	**Intervention (*n* = 78), *n* (%)**		**Control (*n* = 71), *n* (%)**	
**Sex**				
Male	25 (32.1)		21 (29.6)	
Female	53 (67.9)		50 (70.4)	

**General Medical Scheme (GMS) Card holder**				
GMS	67 (85.9)		65 (91.5)	
Non-GMS	11 (14.1)		6 (8.5)	

**Marital status**				
Single	8 (10.3)		16 (22.5)	
Married	38 (48.7)		34 (47.9)	
Widowed	17 (21.8)		7 (9.9)	
Divorced/separated	11 (14.1)		14 (19.7)	
In a relationship	4 (5.1)		0 (0.0)	

**Educational level**				
Primary	29 (37.2)		27 (38.0)	
Some secondary	19 (24.4)		20 (28.2)	
Complete secondary	14 (17.9)		11 (15.5)	
College/university	16 (20.5)		13 (18.3)	

**Employment status**				
Full-time employment	0 (0.0)		2 (2.8)	
Part-time employment	6 (7.7)		1 (1.4)	
Not working due to condition	17 (21.8)		23 (32.4)	
Unemployed	5 (6.4)		5 (7.0)	
Retired	46 (59.0)		37 (52.1)	
Carer	1 (1.3)		2 (2.8)	
Full-time housewife	3 (3.8)		1 (1.4)	

**Mobility aid**				
Independent	57 (73.1)		53 (74.6)	
With aid	15 (19.2)		17 (23.9)	
Wheelchair user	6 (7.7)		1 (1.4)	

**Living situation**				
Living alone	26 (33.3)		18 (25.4)	
Living with family	49 (62.8)		53 (74.6)	
Living with others	3 (3.8)		0 (0.0)	

	**Mean (SD)**	**Median(IQR)**	**Mean (SD)**	**Median (IQR)**

**Age, years**	65.5 (9.3)	66 (15)	65.9 (10.5)	66 (17)

**Mean number of conditions**	4.4 (1.7)	4 (2)	4.7 (2.1)	4 (3)

**Mean number of repeat medications**	9.1 (5.4)	7 (7)	8.5 (5.1)	7 (7)

**Baseline primary outcome measures**				
EQ-VAS	60.6 (19.9)	60 (15)	58.2 (21.3)	60 (35)
FAI total score	25.7 (8.1)	27 (12)	25.1 (7.2)	26 (11)

EQ-VAS = EQ visual analogue scale. FAI = Frenchay Activities Index.

**Table 2.** Adjusted multiple linear regression intention-to-treat analyses for primary and secondary outcomes using complete case analyses

**Outcome**	**Immediate follow-up**			**6-month follow-up**		

	**Mean difference**			**Mean difference**		
	*n*	**(95% CI)**	***P*-value^a^**	***n***	**(95% CI)**	***P*-value^a^**
**Primary outcomes**						
EQ-VAS^b^	124	7.86 (0.92 to 14.80)	**0.027**	121	6.07 (−1.77 to 13.91)	0.127
EQ-5D-3L index score^b^	132	0.04 (−0.06 to 0.13)	0.992	121	0.10 (−0.01 to 0.22)	0.077
FAI^b^	121	1.22 (−0.84 to 3.29)	0.243	121	1.20 (−0.89 to 3.29)	0.257

**Secondary outcomes**						
NEADL scale^b^				121	1.84 (−0.89 to 4.58)	0.184
SEMCD^b^				121	0.52 (0.00 to 1.05)	0.052
HADS-A^b^				121	−0.45 (−1.59 to 0.69)	0.436
HADS-D^b^				121	−0.49 (−1.61 to 0.63)	0.387
COPM-P^b,c^				114	0.75 (−0.07 to 1.57)	0.073
COPM-S^b,c^				114	1.24 (0.43 to 2.06)	**0.003**
GP visits^b^				121	−0.24 (−1.40 to 0.91)	0.676
Emergency visits^b^				121	−0.05 (−0.43 to 0.34)	0.807
Hospital outpatients^b^				121	−1.69 (−2.66 to −0.72)	**0.001**
Hospital visits^b^				121	−0.20 (−0.45 to 0.06)	0.131
Hospital nights^b^				121	−2.87 (−5.92 to 0.19)	0.066

a P-values ≤0.05 are shown in bold.

b Adjusted for sex, baseline score, area, number of conditions at baseline, and age.

c Seven participants did not identify goals in the COPM at baseline, that is, 142 provided COPM baseline data, 114 participants provided COPM data at 6-month follow-up. COPM-P = Canadian Occupational Performance Measure: Performance subscale. COPM-S = Canadian Occupational Satisfaction Measure: Satisfaction subscale. EQ-VAS = EQ visual analogue scale. FAI = Frenchay Activities Index. HADS-A = Hospital Anxiety and Depression Scale: Anxiety subscale. HADS-D = Hospital Anxiety and Depression: Depression subscale. NEADL = Nottingham Extended Activities of Daily Living. SEMCD = Stanford Chronic Disease Self-Efficacy 6-item Scale.

### Primary outcomes

For HRQoL (EQ-VAS), significant differences were seen in favour of the intervention group at immediate follow-up (adjusted MD = 7.86; 95% CI = 0.92 to 14.80; *P* = 0.027). However, there were no differences between intervention and control groups in the EQ-5D-3L index (adjusted MD = 0.04; 95% CI = −0.06 to 0.13; *P* = 0.992) and frequency of activity participation (adjusted MD = 1.22; 95% CI = −0.84 to 3.29; *P* = 0.243) at immediate follow-up. There were no differences in primary outcomes at 6-month follow-up (Table 2). There were no differences between intervention and control groups in the EQ-5D-3L index and frequency of activity participation at immediate and 6-month follow-up.

#### Subgroup analyses of primary outcomes

Pre-planned subgroup analyses examined the effect of age (<65 and ≥65 years) and number of conditions (<4 and ≥4) on primary outcomes (Table 3). Immediately post-intervention, for those aged <65 years, compared with those aged ≥65 years, no evidence of a difference was found in HRQoL (EQ-VAS); however, there was a significant effect in favour of the intervention for frequency of activity participation (adjusted MD = 6.13; 95% CI = 1.93 to 10.34; *P* = 0.005).

**Table 3.** Adjusted multiple linear regression intention to treat and per protocol of primary outcomes using complete case analyses exploring the interaction with age (<65 versus ≥65) and number of conditions (<4 versus ≥4)

**Outcome**	**Intention to treat**				**Per protocol**			
	**Immediate follow-up**		**6-month follow-up**		**Immediate follow-up**		**6-month follow-up**	
	**Mean difference (95% CI)**	***P*-value^a^**	**Mean difference (95% CI)**	***P*-value^a^**	**Mean difference (95% CI)**	***P*-value^a^**	**Mean difference (95% CI)**	***P*-value^a^**
**Age, years**								
EQ-VAS								
Age (<65 versus ≥65)^b^	11.47 (−3.23 to 26.18)	0.125	25.39 (6.81 to 43.98)	**0.008**	8.49 (−6.72 to 23.70)	0.270	(3.19 to 43.06) 23.13	**0.024**
FAI								
Age (<65 versus ≥65)^c^	6.13 (1.93 to 10.34)	**0.005**	4.74 (−0.53 to 10.01)	0.077	4.90 (1.49 to 8.30)	**0.005**	4.98 (−0.34 to 10.30)	0.066
**Number of conditions**								
EQ-VAS								
Conditions (<4 versus ≥4)^d^	−2.68 (−17.25 to 11.88)	0.716	−5.57 (−25.01 to 13.88)	0.570	−3.10 (−18.04 to 11.82)	0.681	−3.01 (−23.94 to 17.93)	0.775
FAI								
Conditions (<4 versus ≥4)^e^	−3.45 (−7.84 to 0.95)	0.123	−0.74 (−6.07 to 4.60)	0.785	−3.48 (−8.15 to 1.18)	0.141	−0.55 (−5.91 to 4.81)	0.839

a P-values ≤0.05 are shown in bold.

b Adjusted for sex, baseline EQ-VAS, area, and number of conditions at baseline.

c Adjusted for sex, baseline FAI total, area, and number of conditions at baseline.

d Adjusted for sex, baseline EQ-VAS, area, and age.

e Adjusted for sex, baseline FAI total, area, and age. EQ-VAS = EQ visual analogue scale. FAI = Frenchay Activities Index.

At 6-month follow-up, in those aged <65 years, compared with those aged ≥65 years, the effect of the intervention compared with usual care was significant for HRQoL (EQ-VAS) only (adjusted MD = 25.39; 95% CI = 6.81 to 43.98; *P* = 0.008).

### Secondary outcomes

Secondary outcomes were examined at 6 months and no evidence of significant differences were seen in activities of daily living (Nottingham Extended Activities of Daily Living scale), self-efficacy (SEMCD), or anxiety and depression (HADS-A and HADS-D) (Table 2). While there was no difference in perceptions of occupational performance (COPM-P), there was a statistically significant difference in perceptions of occupational satisfaction (COPM-S) in the intervention compared with the control group (adjusted MD = 1.24; 95% CI = 0.43 to 2.06; *P* = 0.003). While no differences were found in other elements of self-reported healthcare utilisation, a significant difference was seen in favour of the intervention group in hospital outpatient appointments (adjusted MD = −1.69; 95% CI = −2.66 to −0.72; *P* = 0.001).

Adjusted per protocol analyses found significant differences in favour of the intervention in self-efficacy (SEMCD), satisfaction with and ability to perform activities (COPM-S and COPM-P), self-reported outpatient appointments, and nights spent in hospital (see Supplementary Table S4 for details).

## DISCUSSION

### Summary

This study is the first definitive RCT of OPTIMAL, an occupational therapy self-management support programme for individuals with multimorbidity in primary care.^3^ The programme was effective in improving HRQoL, as measured by the EQ-VAS, at immediate follow-up, although this effect was not maintained at 6-month follow-up, and there was no effect on the EQ-5D index at either time point. The EQ-VAS provides data presenting participant’s self-assessment of their health, while the index score is based on societal preferences for health states.^19^ There was evidence, based on pre-planned subgroup analyses, that the programme was effective for younger participants, aged <65 years, in improving HRQoL. Regarding secondary outcomes, OPTIMAL showed an effect on occupational satisfaction and self-reported hospital outpatient appointments at 6-month followup, but had no effect on all other outcomes.

### Strengths and limitations

This RCT, which investigated OPTIMAL, was based on previous feasibility and pilot studies, as per the Medical Research Council framework for complex interventions, which was a strength, and it was reported following CONSORT guidelines for parallel trials.^10^^,^^11^ The pragmatic nature of the RCT, conducted in Irish primary care settings and using referral processes similar to those used in practice, was designed to include participants representative of those with multimorbidity in primary care. Study retention was high.

Data regarding the number of patients approached by GPs and primary care team clinicians were not collected given the pragmatic nature of the study, reflecting routine service referral pathways. It is therefore not possible to determine an overall response rate or draw definitive conclusions about the programme’s generalisability to individuals with multimorbidity in primary care. However, the participants were a fairly representative group of individuals with complex multimorbidity having an average of four conditions, eight to nine regular medications, and a mean age of 65 years, which is younger than previous multimorbidity trials.^3^^,^^4^

A further limitation is that multiple assessors conducted baseline assessments, however, training was provided in outcome measure administration to minimise the risk of rater bias.^31^ One researcher conducted all 6-month follow-up assessments via interview, blinded to allocation. The original power calculation was revised downwards from 90% to 80% because of recruitment difficulties. This limitation increases the possibility of making a type II error due to an inadequate sample size, that is, a ‘false negative’ finding. Bias as a result of selective outcome reporting is a concern in trial reporting.^12^ The Goal Attainment Scale^32^ was included as a secondary outcome measure in the trial registry. However, in both the pilot trial and the present study, the Goal Attainment Scale was used with the intervention group participants only during intervention to assist in goal setting.^11^ There was no comparison with control group participants.

In the present study there were some inconsistencies in use of the Goal Attainment Scale to guide programme goal setting across sites. As the measure was a secondary outcome measure with no comparison, these results are not reported.

The subgroup analyses were preplanned based on the literature, which recommends targeting of multimorbidity interventions across the age range and evidence suggesting that those with higher levels of morbidity are at risk of poorer outcomes.^3^ While such analyses can be useful in individualising patient care and provide evidence that can guide targeting of interventions, these should be interpreted with caution given the study was not powered to detect these subgroup differences.

### Comparison with existing literature

The updated 2018 meta-analysis of HRQoL from the Cochrane review conducted by the 3D team^4^ included 14 studies with a range of interventions, but found little or no benefit. A 2019 study of a clinical medication review for patients with multimorbidity found no improvements in the EQ-5D index score but, like OPTIMAL, the present study did find improvements in the EQ-VAS.^33^ It is not clear why these interventions have shown significant improvements in the EQ-VAS but not in the index, and there is a need for further consideration of measures used to detect changes in HRQoL (see Supplementary Table S7 for a comparison of OPTIMAL EQ-5D index scores with the 3D trial).

Core OPTIMAL programme components including information provision, problem solving, and goal setting, targeted risk factors and health behaviours. Those with multimorbidity may require more intensive or ongoing interventions to improve outcomes such as HRQoL. While there was an improvement in HRQoL at immediate follow-up, as measured by the EQ-VAS, this was not sustained at 6-month follow-up. The immediate effect on HRQoL may reflect the short-term benefit gained from the programme’s social interaction. Subgroup analyses for patients aged <65 years suggested HRQoL improvements at 6-month follow-up and improved frequency of activity participation immediately post-intervention. Older individuals with multimorbidity may have developed coping strategies over the years,^34^ or may have less capacity to adopt new behaviours. Approaches such as OPTIMAL may be more effective for adults aged 40–65 years with multimorbidity to develop self-management strategies, thus enhancing elements of HRQoL.

The OPTIMAL programme did not have an effect on frequency of activity participation. Previous studies have suggested that interventions may be effective if they target functioning.^3^^,^^35^^,^^36^ While the OPTIMAL programme focuses on improving activity participation and is led by occupational therapists, it covers a wide range of topics, and may not have sufficiently targeted participants’ functional concerns. While subgroup analyses results should be interpreted with caution, it is possible that younger participants with multimorbidity were initially less active in social and community activities, and had more scope for improvement. However, it does not appear that this effect was sustained after programme completion.

There were mixed effects on secondary outcomes relating to function. The significant difference in satisfaction with occupational (activity) performance (COPM-S) is consistent with previous research, suggesting that satisfaction with participation for those with multimorbidity had a greater effect on wellbeing than performance or activity accomplishment.^37^

The OPTIMAL programme was guided by self-efficacy, a concept developed from social cognitive theory by Bandura.^38^ While intention-to-treat analysis found no significant differences in self-efficacy, as measured by the SEMCD, the per protocol analysis found a statistically significant difference for intervention participants at 6-month follow-up. The original RCT of the Stanford chronic disease self-management programme^39^ also reported improved self-efficacy that was associated with improved health status and reduced healthcare utilisation. However, these findings have not been replicated in settings outside the US, and the present study does not provide clear evidence of an impact on self-efficacy.^40^^,^^41^

Patients with multimorbidity have higher levels of mental health problems, which are associated with increased healthcare utilisation, cost, and activity limitations.^42^^–^^45^ No differences were found in anxiety and depression at 6-month follow-up. Only one of the OPTIMAL programme sessions specifically addressed mental wellbeing, which may not sufficiently address anxiety and depression.

Previous studies of chronic disease self-management programmes have found minimal improvements in depression, despite emotional management being a core aspect of effective self-management.^7^

A significant difference was found in self-reported hospital outpatient appointments but not in other elements of healthcare utilisation. Previous studies of self-management programmes produced inconsistent results regarding healthcare utilisation.^46^^,^^47^ OPTIMAL includes strategies for improving communication with healthcare providers and managing multiple medications, and it is possible that the programme resulted in more effective use of healthcare providers. OPTIMAL may have improved the patient’s experience of care like the 3D study,^4^ but this was not measured. However, self-management programmes alone may be insufficient to reduce healthcare utilisation in multimorbidity. Self-reported healthcare utilisation was used because of logistical difficulties in accessing clinical records across multiple sites, and under-reporting can be a limitation.^2^^,^^48^^,^^49^ The authors of the present study plan to publish articles on the process evaluation and economic evaluation, which will add further to interpretation of trial results.

### Implications for research

While this trial of an occupational therapy-led self-management support programme found no effect at 6-month follow-up, subgroup analyses suggested a benefit for younger participants with multimorbidity. There remains a need to develop effective interventions targeting both HRQoL and function for patients with multimorbidity in primary care.

Future research should evaluate in more detail the effectiveness of the OPTIMAL programme in younger individuals with multimorbidity, given that patients in this subgroup appear to be at risk of poorer outcomes and existing multimorbidity interventions tend to focus on older adults.^3^
